# Age and Gender Impact on Heart Rate Variability towards Noninvasive Glucose Measurement

**DOI:** 10.3390/s23218697

**Published:** 2023-10-25

**Authors:** Aleksandar Stojmenski, Marjan Gusev, Ivan Chorbev, Stojancho Tudjarski, Lidija Poposka, Marija Vavlukis

**Affiliations:** 1 Faculty of Computer Science and Engineering, Ss. Cyril and Methodius University in Skopje, 1000 Skopje, North Macedonia; marjan.gusev@finki.ukim.mk (M.G.); ivan.chorbev@finki.ukim.mk (I.C.); striki@striki.ai (S.T.); 2 Faculty of Medicine, Ss. Cyril and Methodius University in Skopje, 1000 Skopje, North Macedonia

**Keywords:** heart rate variability, electrocardiogram, glucose levels, machine learning

## Abstract

Heart rate variability (HRV) parameters can reveal the performance of the autonomic nervous system and possibly estimate the type of its malfunction, such as that of detecting the blood glucose level. Therefore, we aim to find the impact of other factors on the proper calculation of HRV. In this paper, we research the relation between HRV and the age and gender of the patient to adjust the threshold correspondingly to the noninvasive glucose estimator that we are developing and improve its performance. While most of the literature research so far addresses healthy patients and only short- or long-term HRV, we apply a more holistic approach by including both healthy patients and patients with arrhythmia and different lengths of HRV measurements (short, middle, and long). The methods necessary to determine the correlation are (i) point biserial correlation, (ii) Pearson correlation, and (iii) Spearman rank correlation. We developed a mathematical model of a linear or monotonic dependence function and a machine learning and deep learning model, building a classification detector and level estimator. We used electrocardiogram (ECG) data from 4 different datasets consisting of 284 subjects. Age and gender influence HRV with a moderate correlation value of 0.58. This work elucidates the intricate interplay between individual input and output parameters compared with previous efforts, where correlations were found between HRV and blood glucose levels using deep learning techniques. It can successfully detect the influence of each input.

## 1. Introduction

Heart rate variability (HRV) is defined by the heart rate variations caused by the periodic change of heart rhythm over time in the absence of physiological activity, postural changes, and emotional stimuli. This labels HRV as a noninvasive marker of the autonomic nervous system (ANS) function [1].

Several papers presented work on the relation between ANS and HRV [2,3]. Since ANS influences cardiac control, changes in HRV are expected due to an inflammatory response (protection against infection) or ANS blockade. Additionally, increased physical activity [4] or excitement reflects increased heartbeats while oxygen is delivered to the body. Dependencies of ANS on gender are also reported [5]. It has so far been concluded that [6], with aging, ANS responds slower to heartbeat-increasing stimuli (e.g., physical activity), which is one possible reason for the negative correlation between HRV and age.

We have previously shown that HRV parameters can predict glucose levels by noninvasive methods [7]. This has brought the concept one step closer to reality and has once more established point-of-care (POC) HRV measurements as one of the most promising candidates for noninvasive glucose testing. Nevertheless, the actual applicability of the technique depends on the degree to which other influences on HRV, such as age and gender, can be systematically eliminated.

HRV is associated with a high risk of heart disease and death in different age groups [8], and it is exciting to understand its variability to increase its applicability as a diagnostics/prevention tool. HRV parameters vary by multiple factors, including gender and age. This paper shows the correlations between short-, middle- and long-term HRV measurements and those parameters in healthy patients and patients with arrhythmia and diabetes.

To be more accurate in estimating HRV, we need to apply correction factors in calculations. This research’s primary goal is to identify the dependencies of HRV on age and gender to build better glucose classifications in future research.

The time-domain HRV parameters are SDNN, ASDNN, SDANN, NN50, pNN50, and rMSSD, and the nonlinear HRV parameters consist of SD1, SD2, and SD1/SD2. In addition to the standard calculation methods, we implement HRV methods that eliminate the influence of various heart arrhythmias, which prevent proper indication of the ANS performance [9]. The methods are based on the elimination of extreme changes in the NN intervals that differ by more than 15%, calculated by concatenating the clean segments or averaging the corresponding HRV on the complete interval or smaller time intervals (1 h, 30, 20, 10, 5, 2, and 1 min).

Analyzing the individual correlations of gender, age, and heart condition data on the HRV parameters, we use the trained models on a fourth, proprietary dataset. Next to the patient as data mentioned above, this dataset also contains patients’ blood glucose data, more specifically, the averaged 3-month HbA1c levels. We aim to find the dependence function of each HRV parameter using trend analysis and deep learning regression models.

Our focus is on investigating these influences while, for the first time compared with other authors, taking into account a combination of the following:

- Inclusion of healthy subjects and subjects with arrhythmia. A comprehensive overview of related work shows that most research was conducted on healthy subjects. This paper includes research on those who have experienced heart arrhythmia (irregular patterns in the heartbeat or heart rhythm) or are being cared for.
- Inclusion of short-term, medium-term, and long-term HRV. Most researchers investigate only long-term HRV, and we aim to include various sizes of short-term HRV in this research.
- Inclusion of specific HRV calculation methods. In our study, we include particular HRV measurement methods [9] to extract the essential performance of ANS, eliminating the influence of heart damage that might influence the HRV results.

Using the abovementioned approach, we hope to elucidate the origins of discrepancies in the conclusions of some previously published works where one of these factors might not have been considered.

This research aims to present the data distribution and find correlations between HRV parameters and age, gender, and other related health conditions, such as glucose level regulation ability.

Research hypotheses:

- RH1: There is a significant negative correlation between HRV parameters (SDNN, RMSSD) and age, irrelevant of heart arrhythmia.
- RH2: HRV (SDNN, RMSSD) is lower for women than for men, irrelevant of heart arrhythmia.
- RH3: The age- and gender-related decline in HRV (SDNN, SADNN, RMSSD) is more pronounced in patients with heart arrhythmia than in healthy individuals.

Research questions:

- RQ1: What is the difference in the correlation between HRV parameters and age/gender over time for healthy subjects and patients with arrhythmia?
- RQ2: Which HRV parameter is the most representative to estimate age and gender dependence for healthy subjects and patients with arrhythmia?
- RQ3: How does the relationship between HRV parameters and age/gender change in patients with arrhythmia?

The results from this research may bring us one step closer to noninvasive blood sugar measurement with point-of-care (POC) devices and commercial ECG measurement accessories.

## 2. Related Work

This section includes a structured and in-depth discussion of the related works, categorized into subsections: HRV, glucose measurement, and machine learning (ML) methods, elucidating the support they provide for our research hypotheses and the rationale behind our method selection.

### 2.1. Heart Rate Variability

Several research papers address the HRV relation to a combination of age and gender. Age has been consistently shown to be negatively correlated with HRV, albeit minor differences in the amount and persistence of this correlation with age can still be found.

Umetani et al. [10] present a significant negative correlation between aging and HRV parameters (pNN50, SDNN, and rMSSD particularly) with 95% confidence. The authors explain an HRV decline with aging, showing pNN50 as the primary contributor with a rapid decline, while rMSSD and SDNN decline gradually. This study also indicates that HRV decreases slowly with aging and at a different rate in male and female subjects.

A similar pattern of HRV decreases with aging was found to be steeper for men (1.07/year) than for women (0.68/year) (*p* < 0.05) [11] without significant gender difference in the association of heart rate to BMI. Supporting these two works is the research by Jensen et al. [12], who also concludes that HRV parameters are negatively correlated with age.

A cross-sectional survey of 4580 healthy Chinese men and women aged 20–85 years was performed to detect correlations of age, gender, and BMI with HbA1c, which can be derived from and is correlated with HRV [13]. The study shows that glycohemoglobin levels (HbA1c) increased with age among all groups divided into quartiles.

HRV predictability was the focus of research realized by Voss et al. [14]. They show that HRV increases in the elderly subject group (age 50–74 years) compared to the younger subject group (25–49 years) and discuss that significant modifications of the HRV indices in terms of age disappeared within the last two age decades (age range 55–74 years). General dependence on gender for many HRV indices, particularly from FD, STSD, SPPA, IA, ACOR, and AMI (highly significant), was proven in young subjects. It is shown that those dependencies disappear with increasing age. According to HRV analysis methods, the influences of age and gender on HRV indices differed partly, whereas in general, the gender influences were considerably weaker than the age influences.

### 2.2. Correlation Analysis

Several research studies analyze the correlation of HRV between men and women. This was usually conducted based on a subset of the parameters with the highest influence on HRV (SDNN, SDANN, ASDNN, RMSSD, NN50, pNN50, SDNNi, HF, LF). Ramaekers et al. [15] explain that cardiac autonomic modulation, as determined by HRV, is significantly lower in healthy women than in healthy men. HRV difference by gender was also concluded by Antelmi et al. [11], finding that HF, rMSSD, and pNN50 measures were more significant among women compared with men (*p* < 0.05) in all age groups.

Jensen et al. [12] found that women had lower HRV than men, addressing that the SDNN time-domain parameter was lower in women than in men. Although lower HRV was concluded among women compared with men, analyzing all time-domain parameters, only SDNNi decreased significantly (*p* < 0.05) in females. They also took into account frequency-domain parameters, showing that only LF was especially (*p*< 0.05) decreased in females [16]. Interestingly, these gender differences have been shown to diminish after the age of 50 [10].

HRV was also shown to be susceptible to other other factors, both innate and acquired. Obesity and weight loss in correlation with HRV parameters were analyzed by Karason et al. [17]. The study showed that obese subjects had significantly lower overall HRV (SDNN), which was due to a reduction in both long-term HRV (SDANN) and, in particular, short-term HRV (SDNN index). The study covered a weight loss group, showing a significant decrease in heart rate (8% prolongation of mean RR) and an increase in overall HRV (SDNN).

HRV was also analyzed concerning race, and initial research concludes racial differences that show Afro-Caribbean subjects having a lower sympathetic drive than age-matched Caucasians [18,19].

Several studies show an inverse correlation of HRV with heart rate itself [11]. A similar influence of heart rate on HRV has already been demonstrated [20,21].

All studies focus their research on healthy patients. Most of the studies focus solely on long-term measurements, and to our knowledge, only two studies [14,17] analyze the impact of short-term HRV, measuring over periods of less than or equal to 30 min.

### 2.3. Machine Learning Methods for Glucose Measurement

ML techniques have gained significant traction in healthcare, offering powerful tools for analyzing complex medical data. In the HRV analysis context, ML algorithms have been employed to extract meaningful insights from HRV data and improve predictive models. Several studies have demonstrated the effectiveness of ML methods in HRV-based risk assessment and disease diagnosis [22,23].

Several recent studies have investigated using HRV for noninvasive glucose monitoring. Gusev and Poposka [24] used ML and neural network methods to correlate HRV with glucose levels, achieving a mean absolute error of 10.5 mg/dL. This means that the average difference between the predicted and actual glucose levels was 10.5 mg/dL. A mean absolute error (MAE) of 10.5 mg/dL is considered acceptable for noninvasive glucose monitoring.

Avci et al. [25] also used ML techniques to develop a noninvasive glucose monitoring system based on HRV, achieving a mean absolute error of 12.3 mg/dL. This is slightly higher than the error achieved by Gusev and Poposka, but it is still within an acceptable range. Wang et al. [26] used a combination of HRV and ML to develop a system with a mean absolute error of 11.4 mg/dL. This is closer to the error achieved by Gusev and Poposka, and it suggests that combining HRV with ML can improve the performance of noninvasive glucose monitoring. Zhang et al. [27] used deep learning to develop a system with a mean absolute error (MAE) of 10.8 mg/dL. This is the lowest error reported in any of the studies, and it suggests that deep learning is a promising approach for noninvasive glucose monitoring.

The golden standard for glucose monitoring is a blood test, which has an MAE of about 5 mg/dL. However, blood tests are invasive and inconvenient, so there is a need for more accurate and convenient methods of glucose monitoring. Noninvasive glucose monitoring systems with an MAE of 10.5 mg/dL could be a valuable tool for people with diabetes or other conditions that require frequent glucose monitoring. It is important to note that the MAE of a noninvasive glucose monitoring system can vary depending on the individual and the conditions under which the system is used. For example, the MAE may be higher if the person exercises or has certain medical conditions. Additionally, the MAE may improve over time as the system is further developed and refined.

Given the different dependencies of HRV, its use for predictive purposes requires a more profound understanding to determine its baseline to varying ages for both genders. This implies that all parameters must be considered to understand the parameter landscape and the different influences fully. Moreover, focusing on the short- and medium-term measurements is crucial to integrate this technique into POC measurement devices. The practical applicability of this technique for glucose prediction will strongly depend on its relevance not only in healthy individuals but also in those with arrhythmia and diabetes as the most prevalent chronic diseases in concerned patients. It is, therefore, necessary to include data on such patients in this research.

## 3. Methods

### 3.1. Calculation of HRV Parameters

HRV parameters are primarily analyzed in time [28] and frequency domains [1]. Time-domain HRV parameters are calculated for changes in heart rate between successive normal (NN) beats. Frequent-domain parameters mainly concern the amount of energy in the ECG signal within different frequency bands (ranges).

#### 3.1.1. HRV Calculation Methods

There are multiple heartbeat types, which are classified in the following five categories: normal (N), atrial (A), ventricular (V), supraventricular (S), fusion (F), and unclassifiable or paced beat (Q), according to AAMI EC57 [29] or IEC 60601-2-33 standards [30]. The calculation of time-domain HRV parameters is based on the analysis of the beat-to-beat intervals. We also know that HRV variables heavily depend on the heartbeat type [31].

Only sequences of NN intervals without A or V heartbeats are analyzed for HRV calculation [32]. It is proved that all ventricular and supraventricular beats and various types of atrial and ventricular arrhythmia significantly impact the validity of HRV results, so their elimination is essential.

Patients with diagnosed atrial fibrillation (AFIB) [33] are excluded from the dataset. Additionally, measurements with a detected sinus pause or arrest will be excluded from HRV parameter processing.

We have developed a proprietary algorithm to extract only NN intervals from the dataset to obtain meaningful HRV parameters [9,34]. This algorithm avoids all beats categorized in V, S, F, and Q beat types and all those N beats where the NN length exceeds a predefined allowable change specified by a threshold (in this research, we have used a value of 15%). Since the muscle movements and loose contacts of ECG electrodes generate a lot of noise that corrupts the ECG signal, the beat detection may have a minor performance by introduced artifacts or by more extended periods of uninterpreted segments. To reduce this side effect, our proprietary algorithm avoids those ECG segments where an artifact has been detected. The algorithm is used to avoid those ECG segments where an artifact has been detected by applying the NN threshold rule defined by (1), where NN(i) represents the length of the segment between consecutive N beats calculated for a particular detected beat with identification *i*, and *T* is the threshold.

(1) NN(n−1)∗T<NN<NN(n−1)∗(2−T)

Allowable NN intervals do not include small-length sequences specifying that the number of NN intervals should be over a predefined threshold (in this research, we have used a value of 6 breaks). The sequences of allowed NN intervals for the calculation of HRV are then used for the calculation of the overall HRV for the analyzed time frame by two methods [9]:

- Average calculating the average of HRV calculated on these sequences;
- Combined calculated over a concatenated sequence of these allowed NN sequences.

The corresponding HRV parameters will be labeled by a letter A (for average) or C (for combined) preceding the HRV parameter.

#### 3.1.2. Analyzed HRV

Since frequency-domain calculation requires the execution of high-complexity algorithms (for fast Fourier transform or discrete Fourier transform), in this research, we address only the following time-domain HRV:

- **SDNN** the standard deviation of all NN intervals is usually analyzed as a median of the variability. It consists of parts from the sympathetic and parasympathetic nervous systems. The SDNN can be described as the regulation system’s overall variability or total power.
- **SDANN** the standard deviation of the average NN interval for all 5 min periods of the entire recording (higher values indicate increased parasympathetic activity);
- **ASDNN** the average of the standard deviation of all R-R intervals for all 5 min segments in the recordings;
- **RMSSD** the square root of the root mean square of the sum of all differences between successive NN intervals (higher values indicate increased parasympathetic activity);
- **NN50** the number of pairs of successive NN intervals that differ by more than 50 ms in the entire recording (higher values indicate increased parasympathetic activity);
- **pNN50** the percentage of successive intervals that differ by more than 50 ms (higher values indicate increased parasympathetic activity).

#### 3.1.3. Analyzed Time Intervals

HRVs are differentiated by the length of the ECG measurement time interval. The standard size for short-term measurements is 5 min, while the standard for long-term measurements is 24 h [35].

In this research, we analyze more details on the length of the time interval for HRV calculation and categorize the following HRV [9]:

- Short-term for all measurements up to 30 min;
- Medium-term for measurements from 30 min up to 8 h;
- Long-term for measurements from 8 h up to 24 h.

### 3.2. Evaluation Methods

In this section, we describe the methods and metrics employed to address our research questions and hypothesis regarding the impact of various factors on HRV. We utilize a combination of statistical techniques and metrics to assess these relationships effectively.

#### 3.2.1. Statistical Analysis

To analyze the data and answer the research questions, we used the following statistical metrics:

- Pearson correlation evaluates the linear relationship between two continuous variables. A relationship is linear when a change in one variable is associated with a proportional change in the other variable [36]. The Pearson method calculates the *r* coefficient as a positive value corresponding to the cases when the second variable tends to increase with an increase in the first and negative value when the second variable tends to decrease. The higher absolute *r*-value close to 1 means a stronger correlation. The *p*-value is the probability that you would have found the current result if the correlation coefficient were zero (null hypothesis). The correlation is statistically significant if the likelihood is lower than the conventional 5% (*p* < 0.05).
- Spearman correlation evaluates the monotonic relationship between two continuous or ordinal variables. In a monotonic relationship, the variables tend to change together, but not necessarily at a constant rate. The Spearman correlation coefficient is based on the ranked values for each variable rather than the raw data [37].
- If a significant correlation is found between specific parameter values using the abovementioned methods, a regression function can be used to model the trend of the parameter values over time [38]. To test the research question about the effect of age on HRV, we will use a linear regression model.
- The *t*-test is a statistical test to compare the means of two groups [39], and in this paper, we compare the means of the HRV scores of the different data groups (male vs. female, healthy patients vs. patients with arrhythmia conditions). Testing the age and gender effect on HRV (RQ3) with a *t*-test needs normally distributed groups of data in healthy patients.
- Addressing RH3, we use the Kolmogorov–Smirnov (KS) [40] test and Mann–Whitney U [41] test to test the patients where the HRV scores are not normally distributed, as it is a nonparametric test without any assumptions about the data distribution. The KS test is used to evaluate whether the data groups are coming from a different population. The calculation of the correlation coefficients between HRV and age and gender for each group compares the magnitude and direction of the corresponding correlations between the groups.
- Statistical metrics to evaluate regression model performance include mean squared error (MSE), root mean squared error (RMSE), and mean absolute error (MAE). In this research, we develop regression models to find the normal HRV value ranges for different age groups and medical conditions. MSE is calculated as the average of the squared differences between the predicted values and the actual values, and RMSE is the square root of MSE. The average of the absolute differences between the predicted values and the actual values reveals the MAE.
- The evaluation of the ML models is based on the determination of true and false positives (TP and FP) for correct and wrong detections of the positive class, and also true and false negatives (TN and FN) addressing the negative class. The true positive rate (TPR) or sensitivity (recall) is the proportion of positive cases that are correctly identified by the model, and the false positive rate (FPR) is the proportion of negative cases that are incorrectly detected. The positive predictive value (PPV) or precision is the proportion of correctly detected positive cases versus all positive predictions. The number of positive class samples is much smaller than the number of negative class samples, revealing a large class imbalance factor. Therefore, we use the F1 score, calculated as the harmonic mean of TPR and PPV to evaluate the classification models that predict the blood glucose regulation ability based on HRV data. Additionally, we use receiver operating characteristic (ROC) as a graph that presents the dependence of TPR and FPR for different thresholds of the model’s output. The area under the ROC curve (AUC) graph provides an aggregate measure of performance applied for different classification thresholds.

#### 3.2.2. Visual Representation of Results

To analyze and better understand the results, we use several graphical tools for a better explanation of statistical measures.

- Scatter charts [42] will be used for showing the data distribution along with the accompanying dependency function.
- Box and whisker charts [43] will be used for identifying the degree of dispersion (spread) and skewness in the data and pointing out potential outliers.

#### 3.2.3. Datasets

The datasets used in this research contain 30,000 electrocardiogram (ECG) recordings from both healthy subjects and patients with arrhythmia measured on 283 different patients. The HRV data are then extracted out of the raw ECG data, as described in Section 3. The Gluco dataset contains the ECG recordings along with the instantaneous blood glucose level and HbA1c as an indication of a 2-month average glucose level.

- MIT-BIH Normal Sinus Rhythm Database (NSRDB) [44] includes 18 long-term ECG recordings of subjects referred to the Arrhythmia Laboratory at Boston’s Beth Israel Hospital. The subjects included in this database were found to have had no significant arrhythmias. Those patients are treated as normal subjects during this research. The database consists of 5 men, aged 26 to 45, and 13 women, aged 20 to 50. The MIT-BIH Arrhythmia Research Center created this database, one of the world’s most widely used ECG databases. The recordings were made at the Beth Israel Hospital in Boston, Massachusetts, and the subjects were all referred to the Arrhythmia Laboratory for evaluation of suspected arrhythmias. Cardiologists carefully annotated the recordings to ensure that they were free of significant arrhythmias.
- MIT-BIH Arrhythmia Database (MitDB) [45] contains 44 half-hour excerpts of two-channel ambulatory ECG recordings obtained from 44 subjects studied by the BIH Arrhythmia Laboratory. The recordings were digitized at 360 samples per second per channel with an 11-bit resolution over a 10 mV range. Two or more cardiologists independently annotated each record. The subjects were 23 men aged 32 to 89 and 21 women aged 23 to 89. Those patients are included in this research as patients with known arrhythmia. HRV parameters are calculated for all short-term HRVs (up to 30 min). The MIT-BIH Arrhythmia Research Center also maintains this database and contains recordings from subjects with various arrhythmias. The recordings were made at the same sampling rate and resolution as NSRDB, and cardiologists also annotated them.
- European ST-T Database (EDB) [46] consists of 90 annotated excerpts of ambulatory ECG recordings from 79 subjects. The subjects were 70 men aged 30 to 84 and 8 women aged 55 to 71. Each record is 2 h long and contains two signals sampled at 250 samples per second with a 12-bit resolution over a nominal 20-millivolt input range. The European Society of Cardiology created this database containing recordings from subjects with ST-segment and T-wave abnormalities. The recordings were made at a higher sampling rate than NSRDB and MitDB, and cardiologists annotated them.
- Gluco proprietary dataset consists of 143 unique patients aged between 40 and 86 [34]. Patients were hospitalized at the Clinic of Cardiology, and long-term 24 h ECG was measured for each patient along with instantaneous blood glucose level and HbA1c as an indication of a 2-month average glucose level. The average age of the subjects in the dataset was 60, and the standard deviation of age was 10. The patients were 92 men aged 41 to 81 and 51 women aged 40 to 86. The recordings were made at the same sampling rate and the resolution as EDB, and cardiologists also annotated them.
  Diabetic patients are treated with a specific method (diet, medicaments, insulin), and their ability to regulate their glucose level is classified between GD, BD, and ND classes. The criteria for the screening and diagnosis of each glucose regulation class can also be determined on the values of the fasting glucose measurement based on
  – Gluco ND, a group of patients with no diabetes;
  – Gluco GD group includes patients with diabetes and good glucose regulation using diet, medicaments, or insulin;
  – Gluco BD group consists of patients with diabetes and bad glucose regulation, although using diet, medicaments, or insulin.

MitDB consists of only 30 min ECGs, so only the first-time duration method is used. EDB consists of 2 h ECGs, so the 30 min ECGs are calculated with the sliding window approach and one 2 h HRV calculation. The NSRDB and Gluco databases consist of long-term 24 h measurements, so the 30 min, 2 h, and 8 h HRV parameters are calculated with the sliding window approach. The analysis of the number of HRV calculation samples (for all datasets) is shown in Table 1.

### 3.3. Modeling Methodology

We used a linear regression model with a prepossessing layer for data normalization and the dense layer as the regular deeply connected neural network layer. The model with the best performance used the Adam optimizer [47] and the mean absolute error loss function [48] running for 100 training epochs. Age and gender were included as independent variables in our predictive models to assess their potential impact on glucose levels. Age, gender, and HRV are multifaceted variables that can exhibit intricate and nonlinear interactions. These relationships may not be readily discernible through simple descriptive statistics alone. Models allow us to capture and quantify these complex interactions effectively and move towards defining normal HRV values for different age groups.

### 3.4. Performance Evaluation Methodology

The interquartile range (IQR) measures variability by dividing a dataset into quartiles. Quartiles divide a rank-ordered dataset into four equal parts. The values dividing each piece are the first, second, and third quartiles, denoted by Q1, Q2, and Q3, respectively. Q1 is the “middle” value in the first half of the rank-ordered dataset. Q2 is the median value in the set. Q3 is the “middle” value in the second half of the rank-ordered dataset.

Our prediction model identifies Q1, Q2 (avg), and Q3 quartiles for the HRV prediction. The score is then measured with the number of quartiles that coincide with the measured quartiles for the specific age group. Afterward, the model performance is measured by TPR, FPR, PPV, and F1 scores.

**Figure 1:**
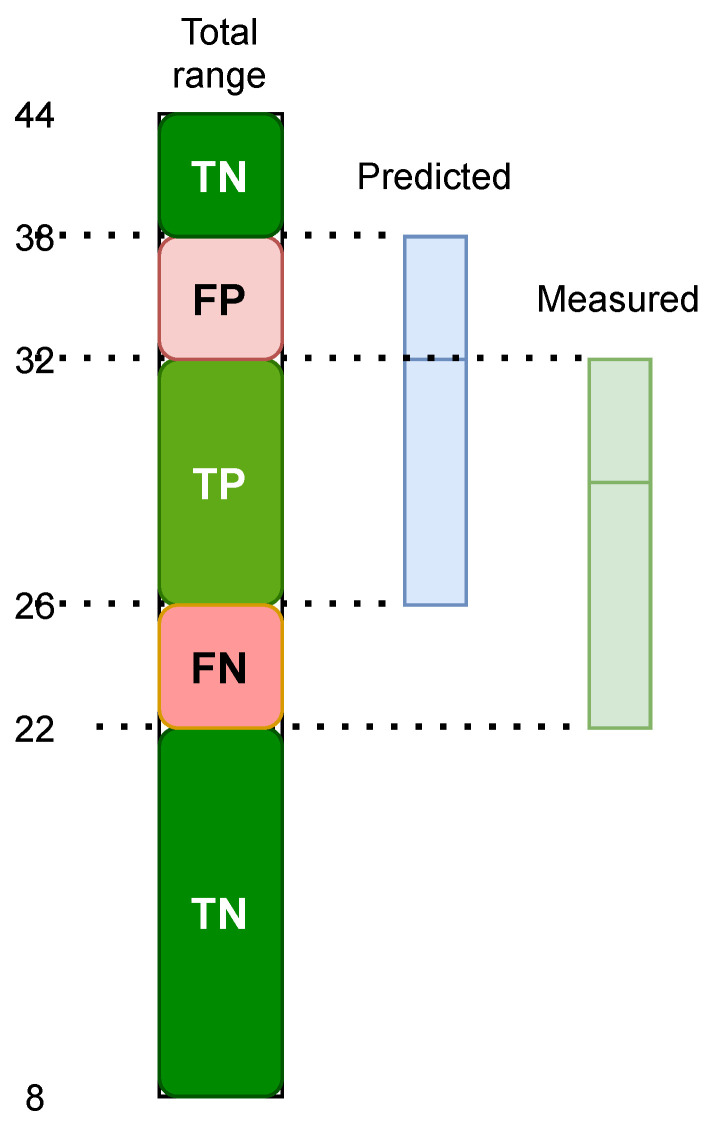
Performance evaluation of predicted range.

To obtain the metrics mentioned above for the model’s performance, we first need to calculate TP, FP, FN, and TN. We use a method based on ranks, where TP is calculated as the correctly predicted rank, and FP is the expected rank but not the measured level. FN is calculated as the rank that was not anticipated but was measured. TN is calculated as the rank that was not predicted and not counted. Figure 1 illustrates an example of the calculations for the evaluation methodology.

### 3.5. Experimental Methodology

HRV parameters are calculated for all benchmark and testing datasets.

The following age groups are analyzed in this research:

- <30 years;
- 30–40 years;
- 40–50 years;
- 50–60 years;
- 60–70 years;
- >70 years.

The data distribution is analyzed for sets of time measurements with different durations, including all analyzed short-, medium- and long-term measurements, including the following time intervals to calculate HRV:

- 30 min;
- 2 h;
- 8 h;
- 24 h.

We use the sliding window method to compare the results obtained from various datasets reasonably. ECG strips are extracted within a window that slides across the overall ECG data stream according to a specified interval. Two variables are considered during this approach: window length (*w*) and sliding offset (*s*). We have used a sliding offset of s=5 min for short- and medium-term measurements and s=10 min for long-term measurements, thereby obtaining more samples to be analyzed in a given period.

The following HRV parameters are analyzed: SDNN, SDANN, ASDNN, RMSSD, NN50, and pNN50, calculated with both the average and combined methods.

Two hundred eighty-eight tests were conducted for six datasets, four duration intervals, six HRV parameters, and two methods. The results are structured in subsections highlighting the influence of gender and age in isolation and, ultimately, the combined influence of both parameters on HRV.

Variations of HRV parameter values are observed for each age group, database, and measurement time interval and shown in box plots to reveal a clear picture of the HRV parameter differences for each of the influence factors.

Scatter plots observe a trend line and the dependency function for the specific plot. Pearson and Spearman correlation coefficients were used to generate the correlation matrices and determine the most representative HRV parameters influenced by age and gender for healthy patients and patients with some of the conditions mentioned above.

F1 score, ROC, and AUC are used as performance metrics for our developed classification ML models. Additionally, MSE, RMSE, and MAE are three metrics used for evaluating the performance of developed regression models that find the normal HRV value ranges for different age groups and medical conditions.

## 4. Results

This section presents the results obtained from the conducted tests of the specified experiments, analyzing them in subsections that cover overall age dependence, gender dependence, and dependence on their health status concerning the autonomic nervous system.

Many images and correlation tables were generated for each conducted testing, analyzing the age and gender aspects and health status. This is why, in this section, we will show only the most important research results.

### 4.1. Age Dependence

**Figure 2:**
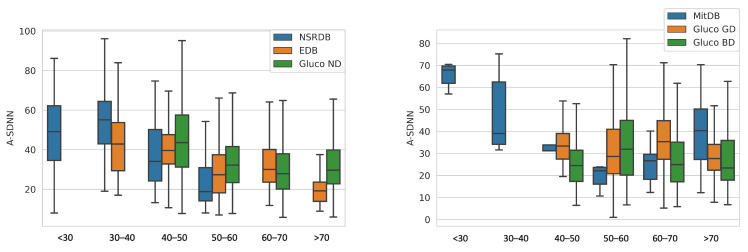
Age distribution of the A_SDNN parameter for healthy patients (**left**) and patients with arrhythmia or diabetes (**right**).

Figure 2 shows the data distribution for the A_SDNN HRV parameter for all datasets, divided by age groups, correspondingly for healthy patients and patients with arrhythmia.

We observe that A_SDNN decreases with aging for healthy patients (EDB, NSRDB, Gluco GD, and Gluco ND). On the other hand, the databases that contain patients with arrhythmia (Gluco BD and MitDB) do not show the same distribution and age dependence.

### 4.2. Dependence on the Average or Combined Calculation Method

**Figure 3:**
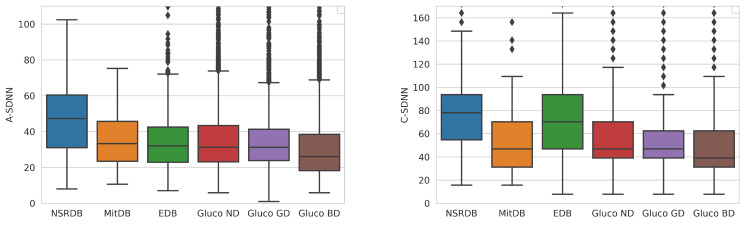
Distribution of the SDNN parameter calculated by the average (**left**) and combined (**right**) methods for the overall dataset. The diamond shapes present the outliers detected in the datasets.

**Figure 4:**
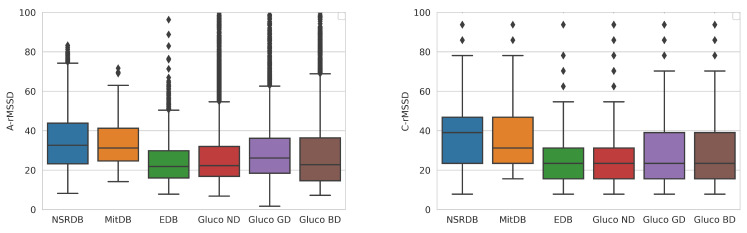
Distribution of the rMSSD parameter calculated by the average (**left**) and combined (**right**) methods for different datasets. The diamond shapes present the outliers detected in the datasets.

Figure 3 and Figure 4 present the differences between the average and combined methods for the calculation of HRV parameters.

The first method is to calculate an average of the calculated HRV within the clean segments of the analyzed ECG measurement. In contrast, the second one is the combined method, which concurs clean segments and calculates one HRV value for the analyzed measurement. The concatenation of clean segments presents an increase in the typical HRV values. Thus, we consider the average method more suitable for calculating age and gender dependency.

Our research has shown that the average method gives more relevant representative results than the combined method. Since the combined method concatenates the HRV calculation into one large HRV measurement, it presents larger values than the actual average. We focus on values calculated with the average method in our further analysis.

### 4.3. Gender Dependence

We observe that females have lower A_SDNN HRV parameters in the MitDB and Gluco (BD, GD, and ND) datasets, which is not the case using the EDB and NSRDB datasets. For the A_rMSSD parameter, females have higher HRV using all datasets except Gluco GD and EDB (although the difference using the EDB dataset is less significant).

**Figure 5:**
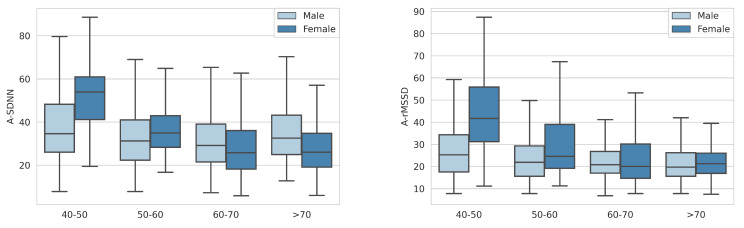
Distribution of A_rMSSD (**left**) and A_SDNN (**right**) for different age and gender groups in the Gluco ND dataset.

**Figure 6:**
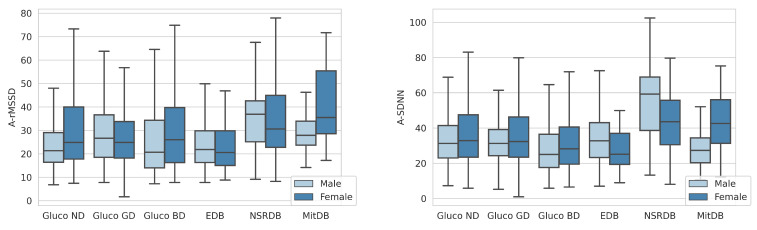
Distribution of A_rMSSD (**left**) and A_SDNN (**right**) versus gender for different datasets.

We observe HRV differences for age and gender groups within the analyzed datasets (Figure 5 and Figure 6) and conclude a trend of lower HRV values for older people. The difference is more noticeable for the A_SDNN parameter.

### 4.4. Age and Gender Dependence Functions

**Figure 7:**
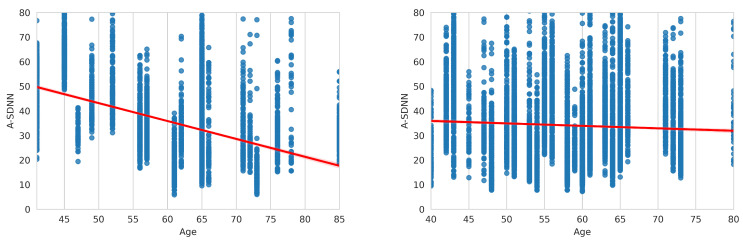
Distribution and dependency function of A_SDNN in the Gluco ND dataset: female (**left**) and male patients (**right**).

Figure 7 presents the dependency function for the A_SDNN parameter using the Gluco ND dataset for male and female patients.

**Figure 8:**
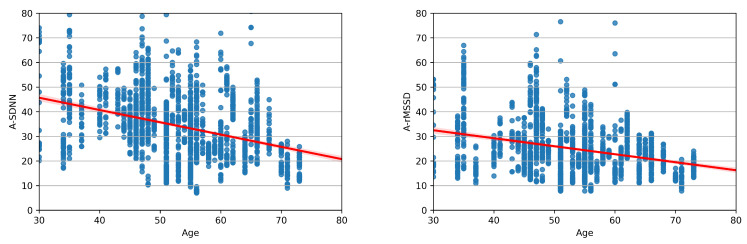
Distribution and dependency function of A_rMSSD (**right**) and A_SDNN (**left**), calculated on the EDB database.

Figure 8 shows the data distribution for A_SDNN and A_RMSSD parameters and the accompanying dependency function. The arithmetic expression of dependency functions will be presented for healthy patients and patients with arrhythmia. The functions are calculated using the EDB database for healthy patients and the MitDB database for patients with arrhythmia.

The dependency function of the A_SDNN parameter for healthy patients (EDB) is calculated by (2). In the following equations, *y* represents the calculated HRV parameter, and *x* represents age, showing the average value for the corresponding HRV parameter.

(2) y=60.62−0.49x

Equation (3) presents the dependency function for the A_rMSSD parameter calculated on healthy patients.

(3) y=42.19−0.32x

The dependency function for the A_SDNN parameter calculated on patients with arrhythmia is shown in Equation (4).

(4) y=44.30−0.13x

Analyzing the patients with arrhythmia, the dependency function for the A_rMSSD parameter is calculated by Equation (5).

(5) y=37.71−0.046x

### 4.5. Prediction Based on Regression Model

This section shows the practical usage of current state-of-the-art deep learning methods for predicting typical HRV values for different age groups and genders. We have built a regression model using Tensorflow 1.13.1 [49] and Keras 2.2.3 [50].

**Figure 9:**
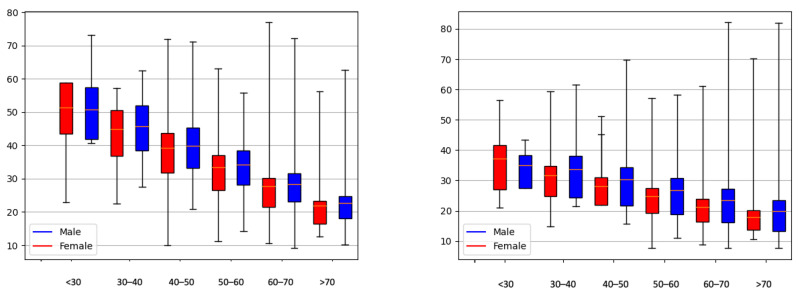
Regression model to predict normal ranges of A_rMSSD (**left**) and A_SDNN (**right**) for different ages and gender.

A preprocessing layer for data normalization and the dense layer as the regular deeply connected neural network layer were included in the modeling process. The inputs for the deep learning model were age group and gender. The model outputs mean, minimum, and maximum value predictions for a given parameter. Currently, the sequential model from Keras supports one value prediction per trained model. Thus, we trained three separate models for the outputs per HRV parameter. The results of the predictions are presented in Table 2 and visualized in Figure 9.

## 5. Discussion

The results presented in this work show the different dependencies and correlations of HRV parameters with age and gender for healthy patients, diabetic patients, and patients with arrhythmia. This section will evaluate the results and perform a correlation analysis.

### 5.1. Statistical Analysis

Several research papers address the connection of HRV parameters with age and gender. A negative correlation of HRV parameters with increasing age is a common conclusion in the literature [10,12,15,20,21].

In this study, we found that males and females have different HRV profiles, as evidenced by the significant differences in the distributions of the HRV parameters A_SDNN and A_rMSSD between the three groups from our database Gluco (Table 3). This finding suggests that gender is an important factor to consider when using HRV to monitor health and well-being.

Our research confirms the negative correlation (stated in Hypothesis 1) between HRV and age. Our research’s best *r* correlation coefficient is the Age/A-ASDNN correlation (r=−0.42, p=0.014), using the EDB database (only healthy patients). The result is similar to the one presented by Ramaekers et al. [15] (r=−0.47, p<0.001) and Umetani et al. [10] (r=−0.41, p<0.05), which are using only healthy subjects. The *p*-value = 0.014 makes this comparison statistically significant.

It is important to note that the HRV dependence on age and gender is also present in patients with arrhythmia (research Hypothesis 3). MitDB (consisting of patients with arrhythmia) shows an inverse correlation for HRV with both age and gender, compared with the other databases that contain only healthy patients. For the gender parameter, this is presented in Table 4, where MitDB has a negative correlation coefficient of r = −0.58 and *p*-value of 0.0018 for A_SDNN. At the same time, the databases for healthy and mixed patients show lower correlations, some of which even reach positive values of up to r = 0.19, with a corresponding *p*-value of approximately *p* = 0.2385. While this correlation was positive, it did not reach statistical significance at the conventional threshold of *p* < 0.05 for healthy patients. We see the same trend in HRV correlations for the age parameter, shown in Table 5. The A_SDANN parameter shows the most significant difference for the patients with arrhythmia, having r = 0.42 with a highly significant *p*-value of approximately *p* = 0.0004, indicating a strong and statistically significant positive correlation in this subgroup. In Table 4, we also see that the patients from the healthy and mixed datasets achieve a negative correlation (r = −0.42, *p* = 0.001) for the A_ASDNN parameter and age, while the MitDB arrhythmia database shows a positive correlation of r = 0.27, *p* = 0.009.

We then performed HRV correlation on each class in our proprietary dataset. The results confirm the abovementioned assumption of inverse HRV dependency for patients with diabetes compared with healthy patients. For HRV/gender correlation, the most significant difference is with the A_SDANN parameter, having r = −0.07 for healthy patients (ND) and r = −0.15 for patients with diabetes with good glucose regulation (GD). The results are shown in Table 6 and Table 7, correspondingly, Pearson and Spearman correlations.

HRV is lower among women than men [11,16]. Our studies confirmed a negative correlation with r=−0.58, *p*-value = 0.0018, for the correlation between gender and A_SDNN using the MitDB database (for patients with arrhythmia).

### 5.2. Deep Learning Models

We have used machine and deep learning methods to develop a classification model to classify three classes, ND, GD, and BD, based on the calculated HRV values specified in this research. We used 10-fold cross-validation to reveal the best-performing model. The features used for training a model are only the HRV measures: A_SDNN, A_rMSSD C_SDNN, and C_rMSSD. The label values are GD, BD, and ND.

Our experiments included tests with 15 different algorithms (Table 8). We used the F1 score for comparison instead of accuracy because of the great class imbalance in our datasets. The F1 score is a more reliable metric for evaluating imbalanced datasets because it takes both precision and recall into account.

**Figure 10:**
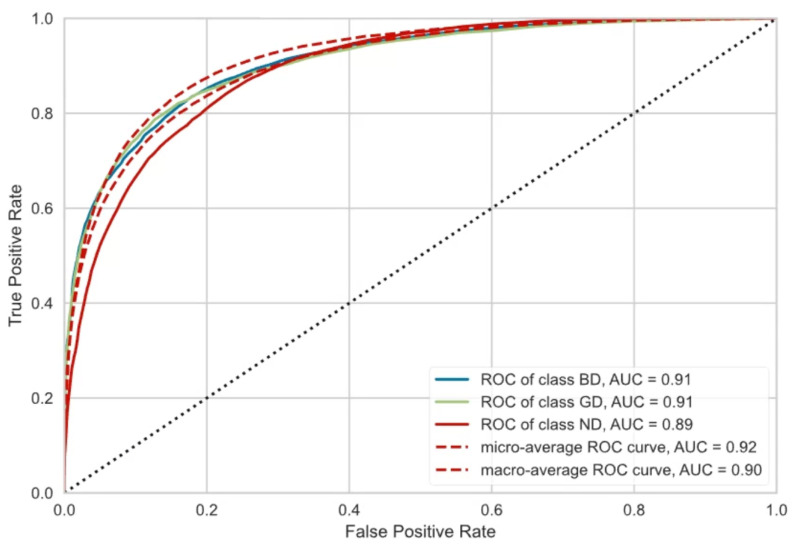
Dependence of HRV on HbA1c: ROC and AUC values.

Figure 10 presents the achieved ROC and AUC values for the extra trees classifier and Table 9 the confusion matrix. According to the F1 scores, the best-performing model is the extra trees classifier, achieving an F1 score of 76.77%. Additionally, the specified model shows the mean squared error (MSE) = 7.15 and the mean absolute error (MAE) = 3.21. The achieved value shows that HRV can determine the diabetes class.

### 5.3. Comparison with Related Work Results

Umetani et al. [10] presented a dependency function for SDNN and age defined with y = 186.9 − 1.5x and rMSSD/age described with y = 32 − 0.69x.

For the rMSSD parameter, we obtained similar yet less gradual results for healthy patients. We can conclude that HRV declines with significantly slower rates for patients with arrhythmia.

For the A_SDNN parameter, we also see differences between healthy patients and patients with arrhythmia or diabetes. The dependency function of age and SDNN for healthy patients rapidly declines, which is not the case for the other patients in the dataset. Compared with Umetani et al., our dependency function for A_SDNN in MitDB also shows a decline but is more gradual. This suggests that the relationship between age and SDNN is different for healthy patients and patients with arrhythmia or diabetes. This inverse coefficient to HRV is also present in the bad glucose regulation group of the Gluco dataset presented above.

Our work extends the work of Benichou et al. [51] by investigating the impact of age and gender on HRV in patients with diabetes. The abovementioned findings suggest that age and gender may play a role in the relationship between HRV and blood glucose levels in patients with diabetes. This is important to consider when developing noninvasive glucose measurement devices based on HRV.

We show a significant improvement of 7% in the random forest supervised classification model for predicting glucose levels [7] on the Gluco dataset using the mean absolute error (MAE) metric, by including age and gender in the parameters list, compared with a baseline model that did not include these variables.

### 5.4. Evaluation of the Research Hypotheses and Questions

Further on, we evaluate the hypothesis and research questions.

- RH1: Section 4.1 showed that older healthy patients have lower A_SDNN and A_rMSSD values than younger patients.
- RH2: Women have lower HRV values than men for the A_SDNN parameter, confirmed by the results from Section 4.3.
- RH3: HRV dependence on age and gender is also present for patients with arrhythmia rather than only for healthy individuals, confirmed by the realized correlation analysis. The age- and gender-related decline in HRV (SDNN, SADNN, RMSSD) is more pronounced in patients with heart arrhythmia than in healthy individuals as seen from the statistical analysis in Section 5.1. Patients with arrhythmia show inverse correlation coefficients for the A_SDNN, A_rMSSD A_ASDNN, A_SDANN, A_NN50, and A_pNN50 parameters, which means that they need to be systematically addressed when predicting blood glucose regulation ability.

- RQ1: There is a negative correlation between HRV (A_SDNN and A_rMSSD) and age for healthy patients and patients with arrhythmia. The corresponding dependence functions are presented by Equations (2)–(5).
- RQ2: SDNN is the most representative HRV parameter (followed by rMSSD) to estimate age and gender dependence, confirmed by the experiments on all databases of our study.
- RQ3: In patients with severe heart conditions, the correlation between the SDNN and RMSSD parameters was significantly weaker than in patients with no or mild heart conditions, as seen in Table 6 and Table 7. This suggests that patients with severe heart conditions have less variability in their heart rate, which can be a sign of autonomic dysfunction. The potential implications of these findings are that HRV parameters could be used as a biomarker for severe heart conditions.

As mentioned above, the results identify the SDNN parameter as the most representative of the distinction between healthy patients and patients with arrhythmia or diabetes. Furthermore, the SDNN parameter is also most dependent on age and gender compared with other HRV parameters.

## 6. Conclusions

Given the different dependencies of HRV, its use for predictive purposes requires understanding its normal range with age and gender differences. This research shows those differences moving towards accurately defining average HRV parameter values.

In this research, we analyzed six HRV parameters over four different datasets, three of which have been (thoroughly) studied in the literature (SDNN, rMSSD, NN50). We also included a proprietary database (GLUCO) that included average blood sugar levels (HBA1c).

### 6.1. Paper Contribution

In all three databases that have been analyzed by other research groups (EDB, MitDB, NSRDB), by including the gender and heart condition data for the patients in the dataset, we found previously undiscovered correlations between some HRV parameters and gender, age, and heart medical conditions. The correlations were also notably present in the Gluco database, with the highest Pearson correlation coefficient between HRV and age of r = 0.42, *p*-value = 0.0004. Furthermore, comparative analysis between the correlations above in each dataset revealed significant differences in the HRV parameter correlation in the MitDB database (r = −0.48, *p*-value = 0.0018), as it solely comprises patients suffering from severe heart conditions. We have successfully identified these differences, enabling us to correct these input parameters in the analyses of the proprietary Gluco database and build better glucose estimation models. In patients with severe heart conditions, the correlation between the SDNN and RMSSD parameters was significantly weaker than in patients with no or mild heart conditions. This suggests that patients with severe heart conditions have less variability in their heart rate, which can be a sign of autonomic dysfunction. The potential implications of these findings are that HRV parameters could be used as a biomarker for severe heart conditions. By tracking changes in HRV parameters over time, doctors could identify patients at risk of developing severe heart conditions. Additionally, HRV parameters could be used to monitor the effectiveness of treatment for severe heart conditions.

We have also successfully identified strong correlations between other HRV parameters and blood sugar levels. Using this, we could predict and categorize patients into three groups according to the predicted blood sugar levels: (i) no diabetes, (ii) diabetes and good glucose regulation using medicaments, and (iii) diabetes and bad glucose regulation despite medicaments.

The primary contribution of our research lies in the comprehensive analysis of HRV parameters across different datasets, encompassing healthy individuals, diabetic patients, and patients with arrhythmia. Our key takeaway is the identification of distinct HRV patterns associated with different health conditions and demographics. In the results section, we have demonstrated that HRV parameters exhibit statistically significant correlations with age and gender, in both healthy subjects and those with medical conditions. Furthermore, we have delineated the typical HRV values for specific demographic groups, shedding light on the expected variations. Our research offers a novel perspective on HRV parameter values, underlining the importance of considering demographic factors in their interpretation. This information has implications for clinical practice, as it can aid in detecting anomalies in HRV patterns early and contribute to more precise diagnostic and prognostic assessments. Furthermore, this research opens a promising route to bringing noninvasive blood sugar level measurements one step closer to end users, as it can be used with ECG data acquired with any modern tracking monitor, including smartwatches, wristbands, and ECG alert devices with >125 Hz measure rate, which opens the capability of obtaining a new measurement of blood sugar every 30 s. This would enable early detection of blood glucose level disbalance and proactively alert potential patients about a condition that could be diabetes related. Patients with arrhythmia show inverse correlation coefficients for the A_SDNN, A_rMSSD HRV parameters, which is very important for blood glucose regulation. We also concluded that the presence of heart arrhythmia is a very important factor to consider when using HRV to estimate blood glucose regulation ability.

### 6.2. Challenges for Future Research

This section discusses the potential practical applications of our findings in healthcare or individual contexts, along with addressing potential limitations and challenges for future research or applications. The following list presents practical applications of HRV for noninvasive glucose measurement in healthcare or individual contexts:

- Wearable devices: HRV can be used to develop wearable devices that can track glucose levels in real time. This could be a valuable tool for people with diabetes who want to have more control over their condition.
- Monitoring treatment efficacy: HRV can be used to monitor the efficacy of treatment for certain diseases, such as heart failure and arrhythmias. This can help doctors to ensure that patients are receiving the correct treatment and that the treatment is working effectively.
- Personalized medicine: HRV can be used to personalize medicine, which is the practice of tailoring medical treatment to the individual patient. This can be performed by using HRV to identify patients at risk for certain diseases or by using HRV to monitor treatment response.
- Early warning system: HRV can be used to develop early warning systems for hypoglycemia and hyperglycemia. This could help people with diabetes to avoid serious complications, such as diabetic ketoacidosis and coma.

On the other hand, the following list shows some potential limitations or challenges for future research:

- Other factors can also affect both HRV and glucose regulation ability, such as smoking, the presence of dyslipidemia, the level of physical activity, the duration of diabetes, the therapy received for existing diseases, and the presence of other diseases.
- HRV data analysis and conversion can be challenging: As described in the methodology section, HRV calculation out of raw ECG data and analysis can be challenging and requires substantial computing power, specialized equipment, and expertise. This can limit the availability of HRV data, and it can make it difficult to use HRV findings in clinical practice.
- HRV is not a perfect biomarker for glucose: HRV is not a perfect biomarker for glucose, and it can be affected by several factors, such as stress, anxiety, and physical activity. It is important to interpret HRV findings in the context of other clinical information, such as blood glucose levels.

Despite these limitations, HRV is a promising biomarker with the potential to improve noninvasive glucose monitoring. Continued research in this area is likely to lead to new and innovative applications of HRV for this purpose.

## Figures and Tables

**Table 1:** Number of samples and patients in the datasets.

Dataset	Patients	Samples (Number of ECG Recordings)			
		30 min	2 h	8 h	24 h
EDB	90	1849	821	/	/
MitDB	44	160	/	/	/
NSRDB	18	2702	2046	1960	1480
Gluco	143	27,040	16,368	15,088	13,352
Gluco BD	45	8468	5151	4748	4202
Gluco GD	22	5177	2518	2321	2054
Gluco ND	76	13,395	8699	8019	7096

**Table 2:** Results from the regression model that predicts HRV versus age and gender.

Age and Gender	A_SDNN Q1	A_SDNN Q3	A_SDNN MEAN	A_rMSSD Q1	A_rMSSD Q3	A_rMSSD MEAN
<30 F	43.50	58.91	51.37	27.10	41.59	37.18
<30 M	41.92	57.48	50.70	27.52	38.29	35.06
30–40 M	36.82	50.64	44.95	24.75	34.67	31.61
30–40 F	38.41	52.07	45.63	24.33	37.97	33.73
40–50 M	31.73	43.80	39.21	21.98	31.06	28.16
40–50 F	33.31	45.23	39.89	21.56	34.36	30.28
50–60 M	26.63	36.97	33.47	19.21	27.44	24.71
50–60 F	28.22	38.40	34.14	18.79	30.74	26.83
60–70 M	21.54	30.13	27.72	16.44	23.83	21.26
60–70 F	23.12	31.56	28.40	16.02	27.13	23.38
>70 M	16.44	23.29	21.98	13.68	20.22	17.81
>70 F	18.03	24.72	22.66	13.25	23.52	19.93

**Table 3:** Statistical tests for different groups in the Gluco database.

Target	DB_A	DB_B	G_A	G_B	S_A	S_B	U_S	U_P	T_S	T_P	KS_S	KS_P
A-SDNN	ND	BD	F	F	3540	2743	5,678,018	0	10.213	0	0.153	0
A-SDNN	GD	BD	F	F	1482	2743	2,345,151	0	7.292	0	0.135	0
A-SDNN	GD	BD	M	M	3594	4960	11,035,986	0	13.496	0	0.248	0
A-SDNN	ND	BD	M	M	7318	4960	22,362,856	0	18.516	0	0.206	0
A-rMSSD	BD	ND	F	M	2743	7318	12,105,515	0	15.128	0	0.263	0
A-SDNN	ND	BD	F	M	3540	4960	11,720,717	0	23.948	0	0.257	0
A-SDNN	GD	BD	F	M	1482	4960	4,802,296	0	15.554	0	0.226	0
A-rMSSD	ND	BD	F	M	3540	4960	10,615,557	0	9.411	0	0.215	0
A-rMSSD	GD	ND	F	M	1482	7318	6,612,967	0	9.844	0	0.205	0
A-rMSSD	GD	BD	F	M	1482	4960	4,277,869	0	6.894	0	0.193	0
A-SDNN	ND	GD	F	M	3540	3594	7,121,736	0	11.820	0	0.136	0
A-SDNN	GD	ND	F	M	1482	7318	5,920,460	0	6.109	0	0.114	0
A-SDNN	ND	ND	F	M	3540	7318	14,405,134	0	9.860	0	0.084	0
A-SDNN	GD	GD	F	M	1482	3594	2,927,992	0	7.701	0	0.160	0
A-SDNN	BD	BD	F	M	2743	4960	7,803,214	0	10.365	0	0.147	0
A-rMSSD	ND	ND	F	M	3540	7318	16,204,281	0	15.140	0	0.192	0
A-rMSSD	BD	BD	F	M	2743	4960	7,975,730	0	10.250	0	0.152	0
A-rMSSD	GD	BD	M	M	3594	4960	10,486,650	0	8.657	0	0.183	0

Legend: F stands for Female, M stands for Male, G_A stands for Gender_A, G_B stands for Gender_Bm, S_A stands for Sample_A, S_B stands for Sample_B, U_S stands for U_Statistic, U_P stands for U_P_Value, T_S stands for T_Statistic, T_P stands for T_P_Value, KS_S stands for KS_Statistic, and KS_P stands for KS_P_Value.

**Table 4:** Pearson correlation between HRV and gender on 30 min files.

	A_SDNN	A_ASDNN	A_SDANN	A_NN50	A_pNN50	A_rMSSD
EDB	12.71%/<0.001	17.17%/<0.001	7.33%/0.027	9.18%/0.012	10.11%/0.004	2.03%/0.572
NSRDB	19.78%/<0.001	24.16%/<0.001	5.21%/0.103	−6.26%/0.067	5.24%/0.098	1.25%/0.718
Gluco	−5.05%/0.411	5.32%/0.455	2.01%/0.472	0.39%/0.092	−12.32%/0.002	−10.04%/0.010
MitDB	−58.00%/0.0018	−19.15%/<0.001	−27.47%/0.009	−17.11%/<0.001	−27.76%/<0.001	−39.25%/<0.001
	**C_SDNN**	**C_ASDNN**	**C_SDANN**	**C_NN50**	**C_pNN50**	**C_rMSSD**
EDB	8.41%/0.017	8.75%/0.015	5.46%/0.102	−13.83%/0.001	4.35%/0.149	3.00%/0.361
NSRDB	13.74%/0.001	18.15%/<0.001	4.98%/0.118	−14.86%/0.001	2.90%/0.374	3.71%/0.288
Gluco	−6.43%/0.049	−3.95%/0.221	−6.70%/0.041	−3.34%/0.320	−12.12%/0.002	−8.58%/0.016
MitDB	−30.60%/<0.001	−31.05%/<0.001	−19.59%/<0.001	16.69%/<0.001	−26.73%/<0.001	−34.61%/<0.001

**Table 5:** Spearman correlation between HRV and age on 30 min files.

	A_SDNN	A_ASDNN	A_SDANN	A_NN50	A_pNN50	A_rMSSD
EDB	−39.47%/0.002	−42.05%/0.004	−12.51%/0.228	4.30%/0.432	−14.14%/0.014	−29.63%/0.01
NSRDB	−29.10%/0.007	−21.61%/0.027	−19.18%/0.053	−2.65%/0.804	−21.63%/0.027	−23.86%/0.042
Gluco	−14.69%/0.01	−13.96%/0.016	−8.69%/0.484	−8.68%/0.347	−12.60%/0.213	−5.77%/0.572
MitDB	3.90%/0.038	27.57%/0.009	42.53%/0.002	−11.98%/0.03	−8.98%/0.047	9.06%/0.014
	**C_SDNN**	**C_ASDNN**	**C_SDANN**	**C_NN50**	**C_pNN50**	**C_rMSSD**
EDB	−24.12%/0.043	−34.33%/0.001	−7.33%/0.456	−3.71%/0.731	−29.17%/0.066	−29.79%/0.011
NSRDB	−31.14%/0.009	−31.65%/0.008	−18.42%/0.066	9.36%/0.731	−18.82%/0.334	−23.69%/0.062
Gluco	−6.95%/0.049	−8.83%/0.221	−3.75%/0.035	2.13%/0.433	−10.51%/0.076	−2.98%/0.031
MitDB	11.59%/0.036	1.40%/0.012	17.83%/0.009	−12.36%/0.01	4.47%/0.031	9.79%/0.001

**Table 6:** Pearson correlation between HRV and age on Gluco dataset, with the classes BD, GD, and ND.

	A_SDNN	A_ASDNN	A_SDANN	A_NN50	A_pNN50	A_rMSSD
ND	−6.00%/0.03	13.59%/0.04	7.30%/0.484	6.54%/0.274	−5.96%/0.062	−13.99%/0.232
BD	−9.17%/0.369	−7.72%/0.793	1.13%/0.566	4.14%/0.735	−19.05%/0.117	−13.49%/0.218
GD	−6.57%/0.595	−8.92%/0.957	−15.89%/0.874	−8.99%/0.874	−6.06%/0.274	3.00%/0.116
	**C_SDNN**	**C_ASDNN**	**C_SDANN**	**C_NN50**	**C_pNN50**	**C_rMSSD**
ND	−8.51%/0.232	−7.10%/0.52	−6.60%/0.484	−4.81%/0.274	−10.35%/0.062	−12.24%/0.232
BD	−8.92%/0.386	−8.22%/0.793	−6.85%/0.011	−9.68%/0.218	−18.37%/0.663	−12.89%/0.008
GD	−14.40%/0.218	−5.78%/0.484	−19.37%/0.957	12.48%/0.043	−1.86%/0.009	2.90%/0.116

**Table 7:** Spearman correlation between HRV and age on the Gluco dataset, with the classes BD, GD, and ND.

	A_SDNN	A_ASDNN	A_SDANN	A_NN50	A_pNN50	A_rMSSD
ND	−21.94%/0.0001	−19.81%/0.0001	−7.02%/0.2849	−10.94%/0.0745	−24.69%/0.0002	−16.04%/0.0002
BD	−10.33%/0.0009	2.86%/0.0965	−7.62%/0.566	−3.55%/0.735	−19.05%/0.286	−13.25%/0.0017
GD	−7.61%/0.0204	−0.72%/0.6721	1.75%/0.484	−13.57%/0.4563	12.67%/0.3408	8.21%/0.1911
	**C_SDNN**	**C_ASDNN**	**C_SDANN**	**C_NN50**	**C_pNN50**	**C_rMSSD**
ND	−7.05%/0.2849	−12.13%/0.0345	−0.53%/0.0345	−18.43%/0.0096	−22.46%/0.0002	−11.68%/0.0428
BD	−12.46%/0.0595	−9.95%/0.0318	−14.08%/0.0173	28.38%/0.0017	−13.67%/0.0002	−12.31%/0.0017
GD	−6.40%/0.0204	−3.07%/0.6721	−8.09%/0.957	12.60%/0.043	5.20%/0.008	10.63%/0.116

**Table 8:** Performance of developed models.

Model	Accuracy	AUC	Recall	Prec.	F1
Extra Trees Classifier	77.40%	89.76%	71.28%	77.45%	76.77%
Random Forest Classifier	75.49%	88.30%	69.06%	75.42%	74.78%
K-Neighbors Classifier	72.04%	84.58%	67.51%	71.74%	71.80%
Decision Tree Classifier	66.37%	72.13%	62.68%	66.45%	66.40%
CatBoost Classifier	68.01%	81.47%	58.00%	67.77%	65.98%
Extreme Gradient Boosting	67.38%	80.47%	57.04%	67.26%	65.15%
Light Gradient Boosting Machine	66.21%	79.53%	54.96%	66.01%	63.46%
Gradient Boosting Classifier	62.36%	74.29%	49.53%	61.46%	58.41%
AdaBoost Classifier	58.21%	69.70%	46.16%	55.79%	54.69%
Quadratic Discriminant Analysis	55.33%	64.89%	39.41%	52.12%	47.58%
Naive Bayes	55.70%	65.98%	39.47%	54.08%	47.09%
Logistic Regression	55.28%	64.32%	37.45%	46.21%	45.31%
Linear Discriminant Analysis	54.94%	64.23%	36.82%	47.69%	44.36%
Ridge Classifier	54.95%	0%	36.51%	45.46%	43.75%
SVM-Linear Kernel	49.65%	0%	36.27%	43.76%	38.42%

**Table 9:** Confusion matrix for the extra trees classifier to classify HbA1c classes based on HRV.

True	Predicted Class		
**Class**	**BD**	**GD**	**ND**
BD	4347	225	1716
GD	387	2291	1396
ND	801	365	10,460

## Data Availability

The data presented in this study are available on request from the corresponding author. The data are not publicly available due to ongoing research.
